# Comparison of Three Methods to Assess the Potential for Bushpig-Domestic Pig Interactions at the Wildlife—Livestock Interface in Uganda

**DOI:** 10.3389/fvets.2018.00295

**Published:** 2018-12-18

**Authors:** Ariane Payne, Peter Ogweng, Alfred Ojok, Eric Etter, Emmanuelle Gilot-Fromont, Charles Masembe, Karl Ståhl, Ferran Jori

**Affiliations:** ^1^Department of Zoology, Entomology and Fisheries Sciences, College of Natural Sciences, Makerere University, Kampala, Uganda; ^2^UMR ASTRE CIRAD-INRA, Department of Production Animals Studies, Faculty of Veterinary Science, University of Pretoria, Onderstepoort, South Africa; ^3^Lyon University, CNRS, Lyon 1 University, VetAgro Sup, Laboratoire de Biométrie et Biologie Evolutive UMR 5558, Marcy l'Etoile, France; ^4^Department of Disease Control and Epidemiology, SVA, National Veterinary Institute, Uppsala, Sweden; ^5^UMR ASTRE CIRAD-INRA, Campus International de Baillarguet, Montpellier, France

**Keywords:** interface, bushpig (*Potamochoerus larvatus*), African swine fever, questionnaire, track observations, camera-trap

## Abstract

Bushpigs (*Potamochoerus larvatus*) are considered a nuisance to farmers because of their crop raiding habits. Through their incursions into farmlands, they may interact with free-ranging domestic pigs and potentially cause transmission of infectious diseases such as African Swine Fever (ASF). The role of the bushpig in the epidemiology of ASF is poorly known and one of the gaps of knowledge is precisely the nature of interaction between bushpigs and domestic pigs. Thus, in this study, we investigated the frequency of bushpig visits to crop fields in rural communities where ASF is endemic, at the edge of a wildlife protected area in northwestern Uganda, to better understand the potential for interaction and disease transmission. We used three methods (questionnaires, camera traps, and observations for tracks) to assess bushpig visits to farmland. These methods were implemented concurrently in 28 farms during rainy and dry seasons. The results obtained by each of the three methods were analyzed by generalized linear mixed models. Potential risk factors including crop type, season, and landscape characteristics related to bushpig ecology were tested as explanatory variables. A generalized linear model and the Kendall test were used to compare the results and consistency of the frequency values obtained by the three methods. A high percentage (75%) of interviewed farmers reported visits from bushpigs in 29.6% of assessed crops (*n* = 145), and a frequency of 0.014 +/−0.05 visits per night was obtained through camera-trapping. Bushpig tracks were detected in 36% of sessions of observation. Cassava (*Manihot esculenta*) and groundnut (*Arachis hypogaea L*.) crop fields were the most visited, and these visits were more common during the rainy than the dry season. Distances from crop sites to the boundary of the protected area and to the river also influenced visit frequency. Camera-trapping was the least sensitive method while questionnaires and track observations presented consistent and complementary results to characterize spatial and temporal visits of bushpig into the crop fields. Evidence from our study shows that when used in combination, these methods can provide useful data to improve our understanding of the interactions between bushpigs and domestic pigs at the wildlife-domestic interface.

## Introduction

In many resource-constrained countries, livestock and wildlife may interact due to the weak biosecurity in subsistence farming systems, abundance of wild species populations, and overlap between farmland, forest, and protected areas. This situation may lead to the transmission of multi-host pathogens shared between livestock, wildlife, and potentially humans, affecting the livelihoods of communities (1, 2). The ecology and behavior of wild hosts and their interactions with livestock are often difficult to assess, and as a result poorly understood. This makes the control of cross-species pathogens difficult to achieve. A number of factors, including the ecology of the wild species, their abundance and protection-classification, farming practices, distance to protected area, and landscape features underpin these interactions (3–6). Understanding of interactions that can potentially lead to pathogen transmission at the wildlife-livestock interface is thus of paramount importance to implement effective disease mitigation strategies (7).

African swine fever virus (ASFV) is a multihost pathogen that is able to infect several species of *Suidae* (both domestic and wild), as well as, soft ticks from the genus *Ornithodoros*. This haemorrhagic, contagious, and potentially lethal disease in domestic pigs is endemic and causes severe economic losses in many African countries, including Uganda where the genotype IX and X had been isolated (8–11). The virus may be maintained and spread within a domestic cycle by pig to pig transmission, occurring through direct contact or contact with infected carcasses, pork products or contaminated fomites (12). The warthog (*Phacochoerus spp*.) and the soft tick (genus *Ornithodoros*) are considered the natural reservoir of the ASFV. When present, they are able to maintain the virus solely in the environment within a sylvatic cycle and allow occasional transmission to the domestic pig, most likely through the tick vector, or potentially through ingestion of infected meat (12). The role of bushpigs (*Potamocheorus spp*.) in the epidemiology of ASF has been less documented. That bushpigs may become infected with ASFV is well-established, and their ability to transmit the virus to domestic pigs has been demonstrated experimentally. However, the role that bushpig really plays in the dynamics of ASF in natural endemic settings remains to be determined (13–15). Indirect interactions between bushpigs and domestic pigs have been previously reported in northwestern Uganda where the two species share resources such as water and crops (16).

Among social research methods, the questionnaire, aimed at recovering knowledge from stakeholders such as farmers, hunters, wildlife rangers, or livestock traders, is considered a practical, fast, and cost effective approach to gather information regarding interactions between wild and domestic animals possibly in a large area and over long periods (5, 16–19).

Direct observations of animals or records of presence indicators (e.g., tracks and fecal droppings) have also been used. They are efficient and usually demand limited resources. Nevertheless, they depend on suitable field conditions, trained personnel and are often time-consuming. They rarely allow a continuous monitoring of the animals but may result in a coarse estimation of the spatial and temporal pattern of the interactions (18, 20).

Camera-trap (CT) is a non-invasive tool for estimating interactions at previously selected spots, allowing simultaneous monitoring of several species and providing behavioral information (21–24). The efficiency of this methods significantly depends on the study design (e.g., number of CTs, spacing, and duration of CTs deployment) and the CT performance which can affect the interpretation of the process being sampled (25). Depending on the model and the number of cameras chosen for the survey, the equipment may be costly and the time needed for visualizing, selecting, and analyzing the pictures and results can be highly demanding.

Unlike observations and CT, telemetry does not require preliminary knowledge on the locations of wildlife-livestock interactions. By following the animal movements, this method provides spatial and temporal data on the overlap between the monitored individuals (wild and domestic) and livestock facilities at a fine-scale. This approach yields a substantial amount of data needed to determine and characterize the interface (26–28). However, the number of tracked animals is constrained by the cost of the telemetric devices and the capture of wild individuals for telemetric collar deployment, limiting the inference of the results. In addition, data management and analyses aimed at studying animal interactions from telemetric data require a solid expertise (29, 30).

Therefore, the choice of one or a combination of several methods usually depends on the specific objective of the study. For instance, investigations on wildlife-livestock interactions and disease transmission should take into account the pathogen of interest, the infected hosts, as well as, their ability to transmit the pathogen through different routes. In those cases, the selection of the method may be constrained by the accessibility to the animals, and the inherent requisite resources. Subsequently several studies have previously used different methods in parallel or consecutively to gather complementary or preliminary data at the interface (31, 32).

In this study, we investigated an *a priori* interface between domestic pigs and bushpigs in a farmland located in the vicinity of the largest national park in Uganda and where both species are known to raid crop fields for feed (16). ASF is endemic in domestic pigs in the area and has been detected occasionally also in bushpigs (9, 15). We estimated the frequency of visits of bushpigs to the crop fields as an indirect measure of the potential for interaction with free ranging pigs. We hypothesized that the season, the type of crop, the proximity of the forest, river, and protected area can influence the occurrence of bushpigs in the fields and thus the potential for direct or indirect interactions with domestic pigs. To test these hypotheses, we used three different methods in parallel: questionnaires, buhspig track presence and a CT survey. We eventually compared the results yielded by these different methods carried out concurrently on the same study site. We highlight the advantages and limits of the different methods used to investigate this wildlife-livestock interface.

## Materials and Methods

### Study Site

**Figure 1:**
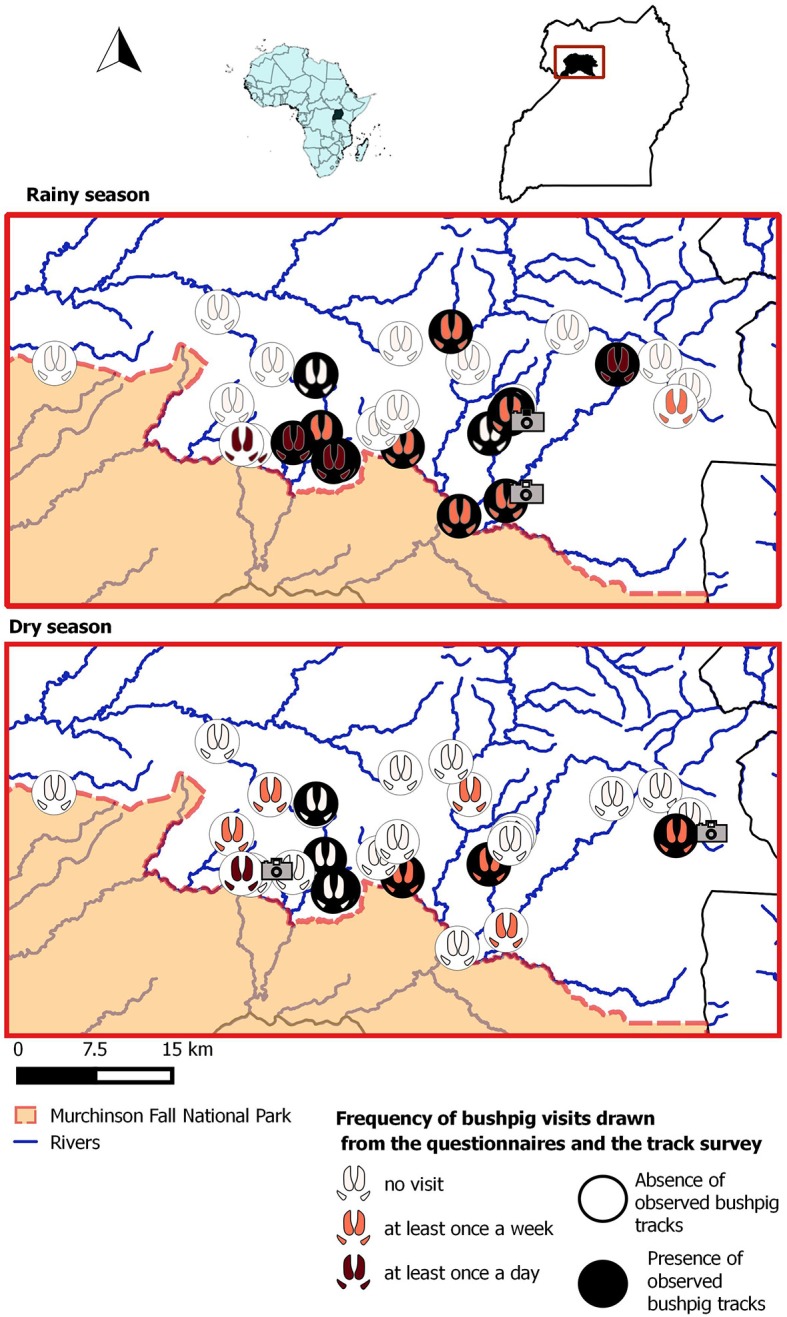
Study site and results yielded by the three methods for each crop field included in the sample for the rainy and the dry season. The camera symbol locates the fields where bushpig visits were detected by CT. Footprints and circles spot the results drawn by the questionnaires and the track observation as described in the legend.

The study was carried out in Nwoya District (total population: 138,500; area: 4,736 km2) located in northwestern Uganda. The study site comprises the northern boundary of an unfenced protected area, the Murchison Falls National Park (NP), and the adjacent rural communities. Specifically, the study included 23 villages of the southern part of the district, covering about half (2,600 km^2^) of the entire district (Figure 1).

The study area is covered by various land items ranging from built up areas, bush, grassland, subsistence farmland and woodland (National Forest Authority). The climate is tropical with a rainy season that runs from April through November; and a dry season from December to March. The area was strategically selected for this study due to recurrent occurrence of ASF outbreaks among a growing free-range domestic pig population (9) and its proximity to an unfenced national park where wildpigs are known to be abundant (16).

### Sample Selection

In order to determine the influence of certain drivers such as land use and distance from the crop fields to the park boundary on the frequency of visits by the bushpigs, we designed a systematic sample scheme based on 54 regular points, each point being spaced 5 km from each other, covering different types of land use and within a distance of 20 km from the Murchison Falls National Park limit. This distance was chosen under the assumption that bushpigs are more abundant inside the park or at the boundary than further away from the park, and that their movement can stretch to a maximum distance of 15 km (33, 34). The regular points were generated by using QGIS 2.10.1.

The spatial selection of the farms in the study was based on the sample scheme. Farms were then selected by convenience to meet different criteria such as agreement of the farmer, type of grown crops, safety for the CT (we avoided crop fields that were not regularly visited by the farmer or exposed to bush fires) and logistic constraints with a target sample size of 30 farms. Twenty-eight farms were finally included in the study. Two farms which were originally part of the sampling scheme declined participation and could not be replaced within the time frame of the study. The total number of crop fields owned by the 28 farmers was 145. The distribution of the different crops owned by the farms is shown in Table 1.

**Table 1:** Type and number of crops grown by the 28 interviewed farmers.

**Type of crop**	**Number**
Cassava	20
Sesame	19
Maize	16
Groundnut	14
Sorghum	14
Rice	14
Soya bean	12
Peas	11
Bean	10
Sweet potato	7
Millet	5
Sugar cane	2
Cabbage	1

The farm was the sampling unit for the questionnaire survey while the crop field was the sampling unit for both the CT and the tracks observations surveys. Within each farm, crop fields to be monitored for the CT and track surveys were chosen to have a balanced sample among the different available crops and the landscape variables. When possible, we monitored the same field during the dry and rainy season. However, some of the crops monitored during the rainy season were harvested at the dry season. In this latter period, we selected, when possible, crops that were not yet harvested within the same farm. As a consequence, some of the crop fields differed between the rainy and the dry season. The distribution of the types of crops that were surveyed according to the seasons is shown in Table 2.

**Table 2:** Distribution of the crops monitored according to the season and their stage (P, planting; Mi, middle; Ma, mature; H, harvested).

**Crop**	**Dry season**	**Rainy season**
Cassava	13 (3 Mi + 10 Ma)	9 (3 Mi + 6 Ma)
Maize	3 (1 Ma + 2 H)	4 (1 Mi + 3 Ma)
Groundnut	1 (H)	3 (Ma)
Sorghum	2 (Ma)	2 (1 Mi + 1 H)
Sweet potato	3 (1 P + 1 Ma + 1H)	1 (Ma)
Soya bean	1 (H)	2 (Mi)
Sesame	2 (H)	1 (P)
Rice	1 (H)	2 (1 Mi + 1 Ma)
Bean	1 (H)	1 (Mi)
Millet	0	2 (1 Mi + 1 Ma)
Peas	1 (H)	0
Sugarcane	0	1 (Mi)
Cabbage	1 (Mi)	0

### Protocol 1: Questionnaires

#### Ethics

Permission to carry out the study was granted by the Ugandan National Council for Science and Technology under the reference number A497. A written consent from the District veterinary officer was obtained prior to the start of any activity in the area. At the time of the interviews, participants were informed that the study was voluntary, confidential, and that they had the choice of ending their participation at any time. An informed consent was given by all participants prior to the implementation of the study.

#### Design and Implementation

The questionnaire, designed for individual interviews, consisted of 115 questions. The questions were designed to collect data related to crop raiding by wild and domestic animals. It was reviewed by a local and international team of epidemiologists and social scientists and uploaded in the KoBo toolbox (KTB) online platform (http://www.kobotoolbox.org/). The interviews were administered in English and translated simultaneously into the local language, Luo, by a trained facilitator fluent in both Luo and English. In order to evaluate the understanding and appropriateness of the questions by all the stakeholders, a pilot trial of seven interviews was carried out in the vicinity of the study area. Subsequently appropriate changes were made in the questionnaire after the trial. The responses and household geolocations were collected using a tablet device (Nexus 9, HTC Corp.).

The questionnaire survey was implemented from November 2016 to January 2017 and farmers were inquired about data corresponding to the dry and rainy seasons pertaining to the year 2016. After having described their farms in terms of crops grown, they were asked about the frequency of visits from wildlife and domestic animals and the season when the visits occurred. We also asked them to rank the species in descending order from the one causing most damage to the one causing the least. Complementary data on the geographical characteristics of the farms were gathered using QGis 2.10. Pisa (Table 3).

**Table 3:** Description of the variables used in the camera-trap and tracks surveys analyses.

**Variable**	**Questionnaire**	**Camera-trap survey**	**Track survey**	**Source**
Response variable: frequency of bushpig visits	Ordinal variable, 5 classes *(transformed in numerical value)* 0: never (*0*) 1: at least once in the cropping season (*2*) 2: at least once a month (*12*) 3: at least once a week (*52*) 4: at least once a day (*365*)	Number of bushpig visits detected per 24 h and per session	Presence of bushpig tracks per session (0/1)	
Explanatory variables	Crop (*5 types*)^a^			ground
	Distance from the crop field to the nearest forest			ground
	Distance from the crop field to the park boundary			NFA/GIS
	Distance from the crop field to the nearest river			NFA/GIS
	Land use (*Bush/grassland/woodland/farmland*)			NFA
	Season (*Dry/rainy*)			Date

a *Grouping of the crops used in the models: cassava/groundnuts/sweet potatoes/maize + millet + sorghum/others: bean + soya beans + rice +sesame + peas + cabbage + sugar cane*.

### Protocol 2: Camera-Trap Survey

We used 6 infra-red motion triggered cameras (Trophy Cam, Bushnell Outdoor Products, USA) which detect movement within a 15 meters range, have a trigger speed of 1 s and display 32 infrared night vision LEDs. The cameras were set to record pictures and 10 s video footages each time a movement was detected within the distance range.

A total of 41 crop fields were selected (mean: 1.46 ± 0.58 crop fields per farm). For each selected crop field, one camera was placed in a site where tracks of wildlife were previously observed by the track observer (see next paragraph). When such observations were absent, the camera was placed where wildlife foragings were more likely to occur, such as the edge of the field bordered by forest or bush, at the opposite end of human settlements. Cameras were tied on a tree or a stalk either at the average bushpig height (~30–50 cm above the ground) or higher (150–200 cm above the ground) with a downward pointing inclination, depending on the surrounding environment. To prevent non-specific triggering of the cameras due to movement of the surrounding vegetation, the grass and branches that fell in the field of view of the CT were removed.

The cameras were set to work continuously during day and night. Date and time were displayed for each photo and video captured. We deployed the cameras from June 2016 to April 2017 in order to cover the rainy (from June to the end of November 2016 and April 2017) and the dry (from December 2016 to the end of March 2017) seasons. We defined a session as a 10 days continuous period of monitoring on the same place with the same camera and position. After each session, the cameras were rotated, so that each crop field was monitored for one session within the rainy season and one session during the rainy season. The location of the camera was recorded using a handheld GPS unit (Garmin GPS Map 60Cx).

All videos and photos were read for species identification and count of the number of individuals. From the photos and the video footages, we recorded the behavior of the animals, such as foraging or just passing through the crop field. We defined independent visits as (1) consecutive photographs or footages of individuals of different species, (2) consecutive photographs or footages of individuals of the same species more than 30 min apart, or (3) non-consecutive photographs or footages of the different or same species (24, 35).

### Protocol 3: Track Observations

Each crop field where the CT was set was investigated twice for wild animal tracks by the same trained field assistant to ensure standardization of the data: once when the camera was set and subsequently, when it was removed at the end of the session. These two observations were recorded during the dry and the rainy seasons as for the CT survey. Each time, the whole area of the field (1.2 acre in average) was scanned to physically look for any animals track. The observer was not the same person as the one watching the video footages from the CT, allowing independence between the two methods. For each observed set of tracks, the species was identified, based on the footprints, droppings, and type of crop damage. When the identification was not possible at the species level, the group (such as “antelope”) was recorded.

### Definition of the Variables

In the questionnaire, the different ordinal qualitative modalities of the frequency of bushpig visit (never/at least once in a cropping season/at least once in a month/at least once in a week/at least once in a day) were transformed into ordinal numerical values (0/2/12/52/365), corresponding to the estimated minimum number of bushpig visits reported per year in the crop field. This was the response variable. The number of visits by bushpigs yielded by CT (quantitative variable) and the presence of tracks (dichotomic variable, 1: present/0: absent) were the response variables for the two other methods, respectively. Given that tracks could remain visible for several days, depending on the seasonal conditions, and that the session was planned to last 10 days, we assigned the value of 1 when the tracks were present either at the first or second observation or both.

The explanatory variables were the season, the type of crop, the distances from the crop field to the nearest forest (we took the distance between the location of the CT and the nearest forest taken by a handheld GPS as described above), to the nearest park boundary and to the nearest river. These two latter distances were calculated between the locations of the camera and these features obtained from administrative shapefiles layers plotted on QGIS. Other variables included land use (bush, grassland, woodland, farmland) obtained from the National Forest Authority (NFA) land cover shapefiles layers (2008) and plotted on QGIS (Table 3). To gain power in the analyses, we merged some of the crops according to their seasonality and assumed similarities in term of palatability of the different crops to bushpigs, drawn from literature and empirical knowledge (see Table 3). The farm was taken as a random variable to take into account the likely dependence of the bushpig visits within one farm.

### Statistical Analysis

From the questionnaire, a descriptive analysis conveying the responses dealing with bushpig visits was carried out. From the CT survey data, we calculated the number of bushpig visits per night per session and the mean and standard error of the visit frequency. From the track observations, we calculated the number of sessions in which the bushpig tracks were observed.

Secondly, we analyzed how the three response variables varied among the type of crop, the distance of the crop field to the forest, park boundary and river; and also between the dry and rainy seasons. We did this by using generalized linear mixed models (GLMM) to take into account the likely dependence within one farm. For the questionnaire and CT survey data sets, we used a Poisson link as usually used for count data having Poisson distribution (36). To standardize survey time among the CT sessions, we used the logarithm of the number of surveillance days per session as an offset. Regarding the track observations, we used a binomial link. We considered the survey time (2 observations on the first and the last day of the session) to be constant among the sessions.

Model selection was performed following the procedure described by Zuur et al. (36). To select the variables to be retained in the fixed part, we started with the most complex model that included all fixed effects. We did not test any interaction as models failed to converge when they were included. We then simplified this starting model by successive steps. At each step, we fitted all possible sub-models. Among the best models (with lowest AICc values) we selected the most parsimonious. The significance of each variable included in the model was assessed using likelihood ratio tests (LRT). The significance of contrasts between categories was assessed using Wald tests. We checked whether overdispersion was present in the residuals of the selected model by calculating the ratio between the sum of squared Pearson residuals and the degrees of freedom. The goodness of fit was assessed by calculating the pseudo R squared giving the conditional (interpreted as variance explained by both fixed and random factors) and marginal (representing the variance explained by fixed factors) coefficient of determination for generalized mixed-effect models. All analyses were performed by using lme4 and MuMIn (37) packages in R 3.4.2 software (38).

### Comparison Among the Different Methods

In order to compare the results from the questionnaire, for which the sampling unit was the farm, with the CT and track survey for which the sampling unit was the crop field, we extracted questionnaire data corresponding to the crop fields monitored by the two other methods. We also transformed the variables corresponding to the visit frequencies given by CT and the questionnaires into dichotomic responses (presence/absence of bushpigs) for each crop field and each season.

We evaluated the degree of agreement between the results obtained by the different methods by computing the Kendall coefficient of concordance for each season, using the irr R package (39).

We also performed a GLMM to investigate the ability of the three methods to detect the bushpig presence in relation to the drivers highlighted by the models selected for each method separately. We did this by including the method (questionnaire/CT survey/track survey) as a qualitative variable in the fixed effects. The response variable was the presence or absence of bushpigs for one crop field with a given method. In order to test whether methods performed differently depending on the situation investigated, we included the interactions between the method and the different drivers found to have an effect on the frequency of bushpig visits. We used a binomial link and the farm was set as a random effect. The selection procedure was the same as already described above.

These analyses were performed using R 3.4.2 with the same packages as described in the previous paragraph.

Finally, we compared the three methods regarding the time and the cost (in terms of manpower and material) that were required to achieve the study. We also compared the characteristics of the data obtained from their collection to their processing leading to the variable of interest.

## Results

### Questionnaires

#### Bushpig Visits

Three quarters of the farmers (21/28) reported visits and crop damage from bushpigs. Among them, only three reported seeing bushpigs in their field during daytime. All the other reports were based on the evidence of bushpig tracks. When asked how they differentiated bushpig tracks from domestic pig ones; six respondents answered that bushpig footprints were bigger than the ones of domestic pigs; five replied that domestic pigs were absent of the area; four that the tracks were in too close vicinity of the bush to be attributable to the domestic pigs; four gave other answers and two were not able to make the difference. No direct interaction was mentioned by any of the respondent.

A total of 43 crop fields, out of the 145 owned by the farmers, were concerned by bushpig visits. Farmers provided different estimates of the visit frequencies depending on the crops they grew. Thirty seven percent of the crop fields (16/43) were visited by bushpigs at least once a day, 58% (25/43) at least once a week, and 5% (2/43) at least once a month.

The selected model for frequency of bushpig visits measured by questionnaires predicted that bushpig visits occurred more often in cassava and groundnut, than in the other surveyed crops. According to this model, the closer the field was to the NP boundary, the more frequent bushpigs forayed into the field (Table 4).

**Table 4:** Models selected to explain the frequency of bushpig visits and presence of bushpig tracks in crop fields yielded by questionnaires, CT or tracks observation.

**Method**	**Response variable**	**Explanatory variable and modality**	**OR and 95% interval**	***P*-value of the Wald test**	**R2m**	**R2c**
Questionnaire	Frequency of	**Crop**				
	bushpig visits	Cassava	Ref			
		Groundnut	0.79 [0.51–1.23]	0.312		
		Sweet potato	0.43 [0.23–0.78]	0.008		
		Maize, sorghum, millet	0.49 [0.33–0.72]	<0.001	0.453	0.616
		Other	0.04 [0.02–0.07]	<0.001		
		**Distance crop field-park boundary**	0.50 [0.27–0.87]	0.015		
Camera-traps	Frequency of bushpig visits	Data did not allow to select a model		
Tracks	Presence/absence of bushpig tracks	**Distance crop field- river**	7.6 [1.72–2540]	0.122		
		**Season**			0.381	0.765
		Dry	Ref			
		Rainy	11.3 [1.69–2286]	0.068		

*For each explanatory variable selected in the models, the table gives the modalities compared, the estimate of odds-ratio with 95% confidence interval and P-value of the Wald test. Marginal (R2m) and conditional (R2c) squared R are given for each selected model*.

#### Visits From Other Species

Among wildlife, the bushpig was the most frequent reported species to raid the crops. This was followed by the African elephant (*Loxodonta africana*) (16/28 farms) and different species of monkeys (11/28 farms), including the black and white colobus monkey (*Colobus guereza*), the vervet monkey (*Cercopithecus aethiops*), and the red tail monkey (*Cercopithecus ascanius)*. The warthog (*Phacochoerus africanus*) was reported by 3 of the farmers. The most frequent reported domestic species to raid the crops was the goat (26/28). Nine farmers reported crop raiding by domestic pigs.

### Camera-Trap Survey

#### Data Collected

Fifty-eight sessions were planned but the data from one of the sessions could not be retrieved due theft of the SD card in the camera. The remaining 57 sessions were distributed as followed: 28 during the dry season and 29 during the rainy season and recorded a total of 692 “camera-days.”

Sessions lasted 12.1 ± 5.2 days on average. The variation in session duration was due to loss of battery power or logistic constraints.

One visit was excluded from the analysis because we could not ascertain the identification of the species.

#### Bushpig Visits

A total of 16 visits from bushpigs were visualized on the pictures and/or video footages, yielding an average frequency of 0.014 visits per day ± 0.05. The 16 visits were distributed among 5 crop fields belonging to 5 different farms, with a range of 1–7 visits per crop field per session. Six visits occurred during the rainy season, (3 in groundnut fields and 3 in cassava fields) while the 10 others occurred during the dry season, in cassava fields (Figure 1). The number of individuals detected on the pictures or footages varied from 1 to 5 with an average of 1.85. We observed bushpigs feeding in the crops in 10 visits and just passing by in the other occurrences.

No model could be selected from the CT results due to a limitation on the amount of data (only 5 fields) (Table 4).

#### Visits From Other Species

Many other wild species were visualized in the pictures and/or video footages, making a total of 75 visits. The most frequent group were the antelopes including dik dik (*Madoqua kirkii*), duiker (*Cephalophus nigrifons*), Uganda kob (*Kobus kob thomasi*), and bushbuck (*Tragelaphus sylvaticus*) with a total of 46 visits. There were no warthogs observed during the study period.

Among domestic animals, goats were the most frequent with 32 visits. This was followed by domestic pigs (19 visits). Eighteen of the visits by domestic pigs occurred during the dry season, and nine were observed in cassava crop fields, eight in a maize crop field and two in a soya bean crop field. The visit occurring in the rainy season was detected in a cassava field, where bushpigs also came. The time interval between the two occurrences was 3 days.

### Bushpig Track Observations

According to the protocol, the number of sessions was the same as for the CT survey with a total of 58 sessions. The mean interval between the two observations of the same crop field was 13.8 ± 4.5 days. Bushpig tracks were detected in 21 of the 58 sessions (36.2%), distributed among 18 different crop fields (out of the 41 monitored) and 15 farms (out of 28). The selected model showed an effect of the distance from the crop field to the nearest river, tracks being more detected in farms located further to rivers, although this effect was not significant. The rainy season tended to be more favorable to the bushpig visits (Table 4). Antelope tracks were the most frequently recorded (31 recordings) among the 58 sessions. Warthog tracks were observed twice in two different fields.

### Comparison of the Results Yielded by the Different Methods

CT detected presence of bushpigs in 9% of the monitored crop fields, whereas the questionnaire and track survey detected bushpig presence in 39 and 37% of the fields, respectively.

The Kendall coefficients assessing the concordance among the three methods were 0.62 (*p* = 0.004) and 0.49 (*p* = 0.06) for the rainy and the dry season, respectively.

To test the hypothesis that methods performed differently according to the situation investigated, we included the interactions between the method and the variables found to have an effect in the GLMM selected for each method, i.e., distance field-park, distance field-river, and season and crop. The crop^*^method interaction was not included as it did not allow to correctly estimate the parameters.

No interaction was retained in the selected model. As for the questionnaire model, bushpigs more often visited cassava, groundnut, and sweet potato fields than the other crops and intruded more frequently in the fields located closer to the park. Similar to the tracks model, bushpig presence was associated with longer distance between the field and the river; and bushpig presence occurred mostly during the rainy season. Questionnaire and track observations reported bushpigs much more often than the CT method, whereas no significant difference was shown by the model between questionnaire and track observations (Table 5). After considering these fixed effects, the variance explained by the random effect (farm effect) was null.

**Table 5:** Model selected to explain the bushpig presence in crop fields, using combined data from three observation methods.

**Response variable**	**Explanatory variable modality**	**OR and 95% interval**	***P*-value of the Wald test**	**R2m**	**R2c**
Presence/absence of bushpigs	**Crop**				
	Cassava	Ref			
	Groundnut	1.77 [0.31–11.42]	0.524		
	Sweet potato	1.39 [0.24–7.48]	0.703		
	Maize, sorghum, millet	0.10 [0.02–0.35]	< 0.001		
	Other crops	0.04 [0.01–0.17]	< 0.001		
	**Distance crop field-park boundary**	0.56 [0.32–0.92]	0.029	0.585	0.585
	**Distance crop field- river**	2.14 [1.37–3.49]	0.001		
	**Season**				
	Dry	Ref			
	Rainy	5.40 [2.12–15.12]	< 0.001		
	**Method**				
	CT	Ref			
	Questionnaire	14.67 [4.25–63.31]	< 0.001		
	Tracks	13.09 [3.80–56.14]	< 0.001		

*For each explanatory variable selected in the models, the table gives the modalities compared, the estimate of odds-ratio with 95% confidence interval and P-value of the Wald test. Marginal (R2m) and conditional (R2c) squared R are given for the selected model*.

The Table 6 provides characteristics such as time, cost required to implement, collect and analyse the data for each of the 3 methods assessed.

**Table 6:** Time and cost spent in euros to implement questionnaire, CT and the track surveys in the 2600 km^2^ study site during one dry and one rainy season, achieve the data collection and analysis.

**Method**		**Questionnaire**	**Camera-trap**	**Track observations**
Required time		***3 months*** including: - design and implementation in Kobo toolbox - Data collection (3 interviews/day in average) - Data analysis	***11 months*** including: - Monitoring and data filtering (10 months) - Data analysis (1 month)	***11 months*** including: - Monitoring (10 months) - Data analysis (1 month)
Estimated cost (for the whole study)		***1625** €* - Manpower: 2 persons (1 researcher and 1 field assistant): 1575 € - Interviewees compensation (Dewormers): 50 €	***4800** €* - Manpower: field assistant 2 days/week to rotate the CT: 500 € - Researcher (supervision and data analysis): 2000 € - CT (x 6): 1800 € - SD cards: 200 € - Batteries: 300 €	***1500** €* - Manpower: field assistant 2 days/week: 500 € - Researcher (supervision and data analysis): 1000 €
Characteristics of the data	Sampling unit	1 farm with at least 1 crop field	1 crop field	1 crop field
	Temporal sampling	Exhaustive	10 days/season	2 observations/season
	Nature of the data Data reliability	Replies to questions Farmer knowledge and memory	Pictures and video footages Field of view of the CT, triggering speed and batteries power	Track observations Observer skills Environment dependence
	Data processing from the data collection to the building of the database	Automatic through Kobo software. Extraction and recoding of the data of interest	1. SD cards collection, pictures downloading, visualization and filtering 2. data of interest entered in a database	1. Data sheets filling 2. Entering data in database
	Variable used to quantify the visit of BP	Number of reported BP visits in the farm's crop fields per year Categorical variable	Number of detected BP visit per day in the crop field Quantitative variable	Absence or presence of BP track in the crop field per session Qualitative variable

*Characteristics of the data collected are also provided*.

From these results, we draw the strengths and weaknesses of each method according to our study. They are summarized in Table 7.

**Table 7:** Strengths and weaknesses of the three methods used to study bushpig visits in crop fields.

**Method**	**Questionnaire**	**Camera-trap**	**Track observation**
Strength	- Cost - Time effective (possibility to gather a large amount of data in a short time)	- Can provide quantitative and behavioral data	- Cost - Adaptive to each crop field
Weakness	- Lack of specificity - Need skills in social science and in local language - Recall bias (retrospective)	- Cost and time-consuming - Lack of sensitivity - Material dependence	- Need skills to identify the tracks - Environment dependence

## Discussion

There are very few scientific publications about bushpigs compared to other species of wild pigs. This is probably due to their nocturnal habits and elusive nature. In this study, we were interested in providing information on the most efficient methods to monitor buhspig incursions into crop fields, as an indirect measure of the potential for direct or indirect interaction with domestic pigs, and thus the potential for disease transmission. We used three different methods (questionnaire, CT, and track survey) to assess visits by bushpigs to crop fields belonging to farms surrounding Murchison Falls NP in North western Uganda. Cassava, groundnut, and sweet potato were the crops associated with the highest frequencies of bushpig visits. Proximity to the NP boundary also had a high correlation with bushpig visits to farms. Bushpigs were more often detected during the rainy season and in fields further away from a river. Consistency between methods was better in the rainy than in the dry season. Questionnaire and track observations reported much more bushpig intrusions into crop fields than CT.

### Drivers of Bushpig Visits

The number of farmers reporting bushpig visits in their crops (75%) are consistent with what was found in a nearby district (Masindi district) by Hill (3) and Tweheyo et al. (5) by using questionnaires. Cassava was also reported as one of the most raided crop by the bushpigs in other studies carried out in Uganda (3–5). Presence of bushpigs in groundnut field has rarely been mentioned except in another study carried out on the same study site (16). This could be explained by a greater availability of this crop in this area and could highlight a feeding preference toward this food resource. Besides, contrary to the tubers such as cassava and sweet potatoes, groundnuts can be dug easily and are available during the rainy season.

The influence of the distance between the farm and the NP boundary suggests that bushpigs might be more abundant at the edge of the park than further away. No data are available regarding the bushpig density outside and inside the park, which could support this result. In Kenya, however, Okoth et al. (40) found that closer distance to a protected area was a risk factor for ASF in domestic pigs linked to potential direct or indirect contacts with bushpigs. More generally, human-wildlife conflicts are more acute alongside protected areas (41).

The effect of the distance to the river is less clear. Bushpigs are known to require access to water and habitat with sufficient moisture to support dense vegetation throughout the year (33, 42). We could therefore have expected that fields closer to rivers would be more visited by bushpigs. Nevertheless, bushpigs might use the riverside during their resting time at daytime whereas crops correspond to feeding sites visited at night, when they are active. Bushpig nocturnal movements can stretch up to 5 km (33, 34) more likely driven by the search of food patches than by water supply. Another explanation could be that distances were calculated to main rivers and not small streams or any other water points such as pond or swamp where bushpigs may also satisfy their need of water. In the same study site, 47% of bushpig sightings by domestic pig keepers occurred in swampy areas and mainly during the dry season (16). It is also possible that the effect of the proximity to the river was influenced by the season, but our data did not allow us to test the interaction between these two variables.

In accordance with our results, Kukielka et al. (16) also reported that crop damages by bushpigs occurred during the wet season when crops are usually ripe. A majority of crops are grown and reach maturity during the rainy season, becoming more attractive for bushpigs. From this point of view, we could suspect a confusion bias between the effects of the crop and the season. However, cassava or sweet potatoes, which were among the most raided crops were also grown during the dry season (see Table 2). We can hypothesize that moisture in the soil during the rainy season makes these tubers easier to dig and thus more palatable for bushpigs.

### Concordance and Contribution of the Three Methods

According to the Kendall coefficient, concordance among the three methods was fairly good during the rainy season and intermediate during the dry season. The fair agreement in the rainy season could be explained by a more accurate assessment of bushpig foray by the farmers in this season due to regular visits in their field to cultivate the crops. Moist soil also makes observation of the tracks more accurate because footprints remain for a longer time and allow a more reliable identification of the species. However, it is worth noting that the selection of the model including the three methods did not result into any effect of the interaction between the method and the season, reflecting that the method efficacy was not influenced by this factor. It is noteworthy that combining the results of the three methods allowed us to obtain a more powerful dataset with better estimation of effects.

Although CT was the most specific method allowing to identify the species with more certainty, visualize the number of individuals and their behavior and provide a quantitative estimation of the bushpig visits, it was also the least sensitive in detecting their presence. The number of visits by bushpigs drawn by the CT might have been underestimated for several reasons. First, we used only one camera per crop field and could have missed bushpig incursions occurring outside the field of view of the camera. Secondly, animals passing rapidly in front of the motion sensor without staying in the field of view may trigger the camera but could not be “captured” even if the trigger speed was set to minimum (1 s), leading to “false negative” results (43). In our study, CTs brought little data and did not allow to characterize the factors affecting bushpig visits in comparison to the two other methods. Increasing the number of cameras would have probably improved the quantity of the data collected by allowing the monitoring of a wider range of the crop field. However, this would have also increased the cost and the time needed to read and analyse the data, when this method was already, by far, the most expensive one (Table 6). This tool may not be appropriate to assess such an interface at this scale and may be more relevant when sites to monitor are restricted to specific small areas [drinking or feeding point for instance, see (21, 24)].

Data recorded from the CTs and the track observations were collected concurrently but independently (i.e., the tracks observer was not aware of the data collected by the camera-trap on the same field and in the same session). We had lower photographic rates than track rates, just as Silveira et al. (44) reported across wild species (including wild pig-like species) when using these methods for wildlife census in Brazil. However, we cannot rule out that the tracks we observed resulted from bushpig visits occurring before the camera-trap was set. This is because the freshness of the tracks can be affected by environmental conditions and therefore it was not possible to accurately date them. It was also difficult to evaluate if tracks resulted from one or several independent visits. Consequently, the main weakness of this method was that it did not provide a frequency in terms of number of visits or number of individuals per time unit. Observations made at more frequent intervals with a cleaning of the previously recorded tracks would improve the accuracy of the method (44), although it would also increase its implementation time and cost. In our study, the track survey was the least costly method, but the quality and the reliability of the data clearly depended on the observer's skill and environmental conditions (Tables 6 and 7).

In the questionnaire study, bushpig forays were more frequently reported by farmers than the results yielded by CT and track surveys. These observations may not be fully reliable since they are based on indirect observations (tracks) and not direct sightings. Moreover, the farmers' ability to identify tracks from bushpigs was heterogeneous and mismatching between bushpigs, warthogs and domestic pig tracks is likely to have occurred, although we cannot say to which extent. Another factor to take into account is that people may be less tolerant toward wildlife than toward livestock regarding crop damages because they have very limited control over wildlife activities (5, 41). Results may thus lack specificity (error by excess) and accuracy (unprecise quantification). They could also suffer systematic errors emanating from lack of completeness of the recollections retrieved (45). Nevertheless, it was one of the least costly and much less time-consuming than the other two methods and it enabled the collection of a large amount of data (Table 6). In addition, questionnaires to farmers allowed us to browse more topics than just visits by pigs in crops. We also gathered local knowledge, perception and strategies practiced to control crop raiding by wildlife that could further be used to propose acceptable and effective measures aiming at limiting the wildlife-livestock interface (31, 46, 47).

Interestingly, the questionnaire and track surveys highlighted different risk factors (type of crop and distance to the park boundary for questionnaire, season and distance to the river for tracks survey). This result reflects the usefulness of the two methods to identify spatial and temporal hotspots for bushpig presence in farmland and the potential for bushpig-domestic pig interaction in our study area. We did not find any interaction between the method and any of the risk factors tested. This means that there was no interference between any of the three implemented methods and the spatial and temporal factors we tested. However, regarding the very low sensitivity and the high cost of camera-trapping in comparison to the questionnaire and track surveys, we would recommend to use one of the two latter methods to study this type of interface.

### Insights Into Bushpig-Domestic Pigs Interaction

Our results showed that bushpigs visit crop fields and that some of these areas may be more at risk of intrusion depending on their location and type of crops grown. Eventhough pig farming is common among the rural community of this area, the domestic pig population is not evenly distributed and is directly related to the distribution of the pigs owners. Mapping the farms keeping domestic pigs would be of particular interest to see how they overlap with bushpigs' preferred field locations, although pig keeping is not a permanent activity and this map would need to be regularly updated. The CT survey detected 19 visits from domestic pigs, most of them (18) occurring during the dry season and nine of them in cassava crop fields. Pigs are usually tethered or housed during the rainy season to prevent them from feeding in the growing crops. This practice may reduce the potential for interactions with bushpigs since we showed that they forayed into crops mostly during the rainy season. Pigs which are not tethered during this season are much more at risk to interact with bushpigs, particularly in cassava fields, which are attractive for the two species. The only observation of consecutive visits by the two species in the same field within a short time interval (3 days) was collected by CT in a cassava field during the rainy season. Our data also showed that domestic pigs might visit crop fields at night, increasing the potential for direct contact with bushpigs. The use of GPS or VHF technology would be of great value to investigate these shared habitats between both species. This tool allows to monitor animal movements at regular and possibly very close intervals, providing a clear picture of the animal's home range and use of the habitat (26–28, 31, 32). Fitting GPS collars on free-ranging domestic pigs and bushpigs in the same area for a long period could provide the spatial and temporal patterns of their habitat-use overlap. In addition, this method may identify interaction spots which may have not been noticed by farmers or by the methods used in this study. However, as bushpigs are elusive and very cautious animals (33, 34, 48). collecting such ecological data can be very challenging and time-consuming (30).

In our study, it was noteworthy that warthogs were not frequently reported or detected in crop fields by any of the implemented methods. This result suggests that crop fields are not a common space of interaction between warthogs and bushpigs or domestic pigs.

## Conclusion

The choice of a method to study wildlife-livestock interface may be constrained by the spatial scale, the accessibility of the animals, the availability of funds, human resources, and time. Here, questionnaires and track observations were most relevant to describe the frequency of bushpig visits and their determinants. The results of this study confirm the interest of using crop field as study area of interaction and pathogen sharing between these two species. Such interactions may lead to the transmission of a number of shared pathogens other than ASFV, such as helminthosis or bovine tuberculosis (48), all affecting the development of the pig sector and impacting the welfare of the rural communities (9, 49) However, other elements are also needed to fully understand the wildlife-livestock interface, such as the functioning of the domestic component (here, domestic pigs). Specific questions will also emerge if the risk of disease transmission is to be analyzed: this step requires taking into account the pathogen of interest, the route of transmission, the kind of hosts infected and their ability to transmit the pathogen. In the case of ASF, future in-depth analyses should focus on the assessment of ASF prevalence levels in the relevant population and the ability to transmit the virus to domestic pigs in this ecosystem.

## Author Contributions

AP and PO designed the study and collected the data in the field with the help of AO who was the local facilitator and the contribution of CM, KS, and FJ. AP, EG-F, and EE performed the statistical analysis. AP wrote the manuscript. AP, KS, CM, and FJ conceptualized the thrust and focus of the manuscript. AP, EG-F, CM, KS, EE, and FJ participated in drafting the manuscript or revising it critically for content.

### Conflict of Interest Statement

The authors declare that the research was conducted in the absence of any commercial or financial relationships that could be construed as a potential conflict of interest.
